# Knowledge of prescribed medication information among patients with limited English proficiency in Sri Lanka

**DOI:** 10.1186/1756-0500-5-658

**Published:** 2012-11-29

**Authors:** Thisara Perera, Priyanga Ranasinghe, Udeshika Perera, Sherin Perera, Madura Adikari, Saroj Jayasinghe, Godwin R Constantine

**Affiliations:** 1 Department of Clinical Medicine, Faculty of Medicine, University of Colombo, Colombo, Sri Lanka; 2 Department of Pharmacology, Faculty of Medicine, University of Colombo, Colombo, Sri Lanka

**Keywords:** Limited English proficiency, Health literacy, Sri Lanka, Cardiac disease

## Abstract

**Background:**

Patients’ knowledge on prescribed medications play a key role in the long term management of cardiac diseases and in determining their outcome. The present study evaluates the knowledge about prescribed medication among cardiac patients and aim to identify factors influencing knowledge.

**Methods:**

A descriptive-cross-sectional study was conducted among 200 adult patients attending clinics at the Cardiology Unit of the National Hospital of Sri Lanka. Knowledge assessment focused on four different sections; drug name, dose, frequency and indication. The total score of 60 was calculated by giving each component the following weighted scores; drug name = 20, indication = 20, drug dose = 10 and frequency = 10. A binary logistic regression analysis to evaluate factors associated with ‘good knowledge’ (total score ≥ 40) was performed.

**Results:**

Among 200 participants 56.5% (n = 113) were males. Mean age was 59.7 ± 8.2 years and a majority (n = 170, 85.0%) were older than 50 years of age. Sinhala was the primary language of 91.5% (n = 183) of participants, while English was the primary language in only two of the study participants (1.0%). Eighty four percent of the participants were educated up to secondary education or above, while 2.5% (n = 5) had no formal education. The overall knowledge (total score-60) on prescribed medications among the study population was ‘poor’ (score ≤ 20) in 46%, ‘adequate’ (score 21–40) in 36.5% and ‘good’ (score ≥ 40) in 17.5%. The results of the binary logistic regression analysis indicates that Secondary (OR-1.53) and Tertiary levels (OR-2.79) of education, self-reported perception of illness as being Moderate (OR-1.23) or Severe (OR-1.70) and being educated by a doctor (as reported by patients) (OR-1.69) significantly increased the odds of having a ‘Good Knowledge of Drugs’. Majority of the patients were unable to read and understand the information written in English. The doctor’s contributed towards educating on drug information only in 33.0% of the patients.

**Conclusion:**

In a resource-poor setting in patients with Limited English Proficiency, lower level of education and misperception of illness severity resulted in reduced knowledge on prescribed medications. Furthermore, being educated by a doctor significantly improved knowledge. However the doctors’ contribution at present to deliver quality health information to their patients was at an unsatisfactory level.

## Background

Choosing a healthy lifestyle, knowing how to seek medical care, and taking advantage of preventive measures require that people understand and use health information appropriately [1,2]. Health literacy (HL) is defined as “The degree to which individuals have the capacity to obtain, process, and understand basic health information and services needed to make appropriate health decisions” [3]. The level of HL in Asia has not been studied in detail though its countries are confronted with a wide range of public health priority issues in which HL can be an important factor. These issues include increasing prevalence of non-communicable diseases, adoption of unhealthy life styles, and emergence of new public health emergencies such as Avian Influenza [4]. Low HL is associated with poor communication between patients and health care providers, and poor health outcomes. It leads to increased rates of hospitalization, improper management of chronic conditions, higher morbidity and mortality rates [5-11].

Knowledge about prescribed medications is one important area of study in the field of health literacy. Studies have shown that poor health literacy is barrier limiting patient’s knowledge on prescribed medications [12,13]. Inadequate knowledge on prescribed medications among patients is also known to be associated with poor compliance [14]. There is evidence suggesting that improved knowledge of the disease condition also improves patient adherence to lifestyle changes and medication [15]. However, most studies on patient’s knowledge about prescribed medications are from western developed countries, while there is a relative lack of research from developing Asian countries.

Sri Lanka is an island in South Asia with a multi-ethnic population, more than 98% of Sri Lankans use either Sinhala or Tamil as their primary language, while only a negligible minority of the population use English as their primary language [16]. The Census of 2001 demonstrated that only 13.2% of the Sinhalese and 24.1% of the Sri Lankan Tamils were able to speak in English [17]. Studies from USA and Sri Lanka have shown that for individuals with Limited English Proficiency (LEP), the inability to communicate in English is the primary barrier to accessing health information and services, thus leading to low HL levels and poor health outcome [3,18]. LEP is associated with increased risks to patient safety, ineffective use of health care facilities and discrimination even in English speaking nations [19-21]. On the other hand patients may not be able to access various other means of health information due to LEP. One such example is the user information leaflets and labels that come with medical tools and drug packages. A study investigating the labeling of drugs and devices in the country found that patients were not aware of the medication and their side effects because the labeling was almost always in English [18]. At present there are no studies exploring factors affecting knowledge on prescribed medications among Sri Lankan patients.

Non communicable diseases are emerging as the foremost health problem in Sri Lanka. Among many non communicable diseases, cardiovascular diseases are increasingly being recognized as an important cause of morbidity and mortality [16,22]. Management of most of the chronic cardiovascular diseases involves the use of long-term treatment with combinations of different drugs [23]. Optimum management of cardiovascular diseases involves many factors other than drugs, of which patient compliance to treatment is one of the most important factors. To ensure good compliance patients should be well informed about the drugs that they are prescribed [24]. Knowledge about potential side effects of a drug is helpful for patients’ in order to recognize side effects early and promptly report to a medical practitioners [25]. We conducted a study to evaluate the knowledge and perception about prescribed medications in a group of patients with cardiovascular disease from Sri Lanka and explored factors determining their knowledge.

## Methods

### Study population and sampling

This descriptive cross-sectional study involving 200 patients was conducted over a period of 6 months at the Institute of Cardiology, National Hospital of Sri Lanka, Colombo (IC-NHSL) from February-August 2008. Data were gathered from patients attending outpatient clinics at IC-NHSL more than once during the past 3 months. Informed written consent was taken prior to data collection. The patients who were too ill to answer the questionnaire and those who did not give consent were excluded from the study. Cardiology clinics are held daily at the IC-NHSL, patients who come to the cardiology clinic are given numbers on the first come basis, and hence in order to choose the sample we used a random sampling technique. In each week we selected a random day to visit the clinic, on each day of study the first patient was chosen randomly from patient number 1 to 10 and then every third patient thereafter was invited for the study. Patients come to clinics usually once per month, we evaluated their knowledge on prescribed medications based on the medications that they were prescribed during the last clinic visit. The interview was thus carried out prior to the consultation with the doctor at IC-NHSL during the present visit.

At the IC-NHSL drugs are delivered to patients’ at its Department of Pharmacy. Each patient receives a Prescription Card (PC) and a Clinic Notes Book (CNB) on which the drug prescription notes are hand written in English by the prescribing doctor. The PC which is retained at the Department of Pharmacy serves as the means of communication between the dispensing pharmacist and prescribing doctor, while the CNB is retained by the patients and brought to hospital during each clinic visit. Drugs issued by Department of Pharmacy at the IC-NHSL do not have printed drug packages (DP) or information leaflets, instead small paper envelope are used for this purpose where necessary drug information is hand written in English by the dispensing pharmacist. The above mentioned documents (DP, CNB and PC) are the written sources of drug information available to the patients. Apart from these, patients may or may not receive verbal information from the prescribing doctors and dispensing pharmacists. Ethical clearance for the study was obtained from the Ethics Review Committee, Faculty of Medicine, University of Colombo and the National Hospital of Sri Lanka, Colombo, Sri Lanka.

### Study instrument

A pre-tested expert-validated interviewer-administered questionnaire containing questions to assess the patients’ socio-demographic data (age, gender, marital status, level of education and present employment status) and knowledge about prescribed drugs (name, doses, frequency and indications) was used as the study instrument. The interview was conducted in patients’ native language (‘Sinhala’ or ‘Tamil’). The questionnaire also contained questions on sources of medical information, communication method employed by doctor/pharmacist and the patients’ ability to read the drug package details. The patients’ PC and CNB were used as the reference to cross check the validity of drug information told by the patients. Patients were asked to categorize the severity of their illness (‘Is your illness mild, moderate or severe?’). The patients were also asked regarding who was involved in educating them (‘Did the doctor educate you about the medication’ or ‘Did the pharmacist educate you about the medication’). We also asked patients how the doctor or pharmacist educated them by using two separate questions (‘Did the doctor/pharmacist educate you; a) only verbally, b) only written or c) both methods’). The clarity of information provided was assessed by asking the patient whether the information provided to them by doctor/pharmacist was clear and understandable; a) drug names, b) drug dose and c) frequency of administration (each section had a separate dichotomous questions e.g. - ‘Was the information provided to you by the doctor on drug dose clear and understandable?’ [Yes/No]). Another question evaluated reasons for the inability understand drug information (‘You were not able to understand drug information due to a) illegible hand writing, b) inability to read English or c) both’). The patients’ perception of their level of knowledge on medications were also evaluated (‘Do you feel that your knowledge on prescribed medications is satisfactory?’).

### Statistical analysis

In the knowledge assessment section, each of the 4 components i.e. name, dose, frequency and indication, was given a score out of 100. When calculating the total score for each component the total number of drugs that the particular patient has been prescribed was also considered. For example a patient who has been prescribed 5 different drugs and correctly knows the name of only 2 drugs, the score was calculated as follows: (100 × 2)/5 = 40 marks. Then a cumulative score out of sixty was obtained for each patient using the four components described above. To calculate this cumulative score (total = 60) each component was given a differentially weighted score depending on relative difficulty in memorization, re-call, and importance as assessed by two independent experts (drug name = 20 points, indication = 20 points, drug dose = 10 points and frequency = 10 points). Then according to this cumulative score the knowledge status was categorized as follows; < 20 ‘poor knowledge’, 21 – 40 ‘adequate knowledge’ and 41 – 60 as ‘good knowledge’. All data were double-entered and cross checked for consistency.

To evaluate factors associated with ‘good knowledge’ a binary logistic regression analysis was performed using the dichotomous variable ‘Good Knowledge of Drugs’ as the dependent factor (0 = No; 1 = Yes). Those who were having a ‘good knowledge’ based on the cumulative score were classified as having a ‘Good Knowledge of Drugs’. The independent co-variants were and age, gender (0 = Male), Level of Education (0 = No formal education), Monthly Income (0 = No income), First Language (0 = Sinhala), Patient perceived severity of illness (0 = Mild), Educated by doctor (0 = No) and Educated by pharmacist (0 = No). For each independent co-variant with more than two categories dummy variables were created. The independent co-variants were selected on the basis of the bi-variate analyses (*r* ≥ ±0.15) to obtain the best fit model. Data were analyzed using SPSS version 15 statistical software package (SPSS Inc., Chicago, IL, USA). The significance of the differences between means was tested using z-test. In all analyses a *P* values < 0.05 was considered statistically significant.

## Results

Among 200 participants 56.5% (n = 113) were males. Mean age was 59.7 ± 8.2 years and a majority (n = 170, 85.0%) were of more than 50 years of age. Sinhala was the primary language of 91.5% (n = 183) of participants, while English was the primary language in only two of the study participants (1.0%). Eighty four percent of the participants had a level of education of either secondary education or above, while 2.5% (n = 5) of the participants had never been to school. Majority of the participants were unemployed or retired (n = 140, 70.0%) and had no adequate regular source of income (n = 87, 43.5%). Table 1 summarizes socio-demographic characteristics of the study participants.

**Table 1:** **Socio-demographic characteristics of the study population (*n*** **= 200)**

	**Number (%)**		
	**All adults**	**Males**	**Females**
Age category			
31 – 40 years	4 (2.0%)	1 (0.9%)	3 (3.4%)
41 – 50 years	26 (13.0%)	20 (17.7%)	6 (6.9%)
51 – 60 years	73 (36.5%)	36 (31.9%)	37 (42.5%)
61 – 70 years	97 (48.5%)	56 (49.6%)	41 (47.1%)
First language			
Sinhala	183 (91.5%)	101 (55.2%)	82 (44.8%)
Tamil	13 (6.5%)	11 (9.7%)	2 (2.3%)
English	2 (1.0%)	0 (0%)	2 (2.3%)
Other	2 (1.0%)	1 (0.9%)	1 (1.1%)
Level of Education			
No formal education	5 (2.5%)	2 (1.8%)	3 (3.4%)
Primary education	27 (13.5%)	13 (11.5%)	14 (16.1%)
Secondary education	160 (80.0%)	93 (82.3%)	67 (77.0%)
Tertiary education	8 (4.0%)	5 (4.4%)	3 (3.4%)
Income			
No certain income	87 (43.5%)	31 (27.4%)	56 (64.4%)
< 15,000 Rupees	77 (38.5%)	53 (46.9%)	24 (27.6%)
≥ 15,000 Rupees	36 (18.0%)	29 (25.7%)	7 (8.0%)
Occupation			
No employment or retired	140 (70.0%)	61 (53.9%)	79 (90.9%)
Professionals	25 (12.5%)	22 (19.5%)	3 (3.4%)
Self-employed	15 (7.5%)	14 (12.4%)	1 (1.1%)
Manual workers	20 (10.0%)	16 (14.2%)	4 (4.6%)

Ninety percent of participants (n = 180) were prescribed more than 3 drugs from the clinic (males – 96.5%, females – 81.6%). Majority of the patients (91.0%, n = 182) were on treatment for cardio-vascular disease for more than one year (males – 92.0%, females – 89.7%). The subjective perception of severity of their own illness was as follows; mild – 8.0% (n = 16), moderate – 42.5% (n = 85) and severe – 49.5% (n = 99).

Table 2 summarizes the sources, mode of delivery and clarity of drug information received by the patients. Accordingly, only 66 patients (33.0%) received drug information by the prescribing doctors, while the dispensing pharmacists had given drug information to all patients. The mode of information delivery by doctors was only verbal in 65 patients (98.5%). The pharmacists gave only written information for 78.0% of participants while 20.5% patients received both verbal and written information. The clarity and understandability of the language used in communicating drug information to the patients were satisfactory (doctors – 100%, pharmacists – 93.0%) (Table 2). It was found that only 55.0% and 52.0% of the patients were able read the drug dose and frequency of administration respectively, while only 1.0% of patients were able to read drug names (Table 3). The main reason for inability to understand drug information written on DP, PC and CNB was their inability to read English (n = 108, 54.0%). A majority of subjects requested further information on the name (n = 191, 95.5%), dose (n = 190, 95.0%), indications (n = 188, 94.0%) and side effects of the drugs (n = 184, 92.0%). The patient’s own perception of their current level of knowledge on prescribed drugs showed that only 38.5% (n = 77) felt their present knowledge was satisfactory. Seventy percent of participants (n = 140) believed that knowledge about drugs they are taking would be helpful for them. Majority of the patients (n = 155, 77.5%) were satisfied with the drug dispensing procedure at IC-NHSL.

**Table 2:** Sources, modes of delivery and clarity of drug information received by patients’

		**Number (%)**		
		**All adults**	**Males**	**Females**
Drug information obtained/given by,				
Prescribing doctor		66 (33.0%)	41 (36.3%)	25 (28.7%)
Dispensing pharmacist		200 (100%)	113 (100%)	87 (100%)
Reading Prescription Card/Clinic Note Book		38 (19.0%)	24 (21.2%)	14 (16.1%)
Family members		16 (8.0%)	7 (6.2%)	9 (10.3%)
General practitioner/family doctor		14 (7.0%)	8 (7.1%)	6 (6.9%)
Modes of drug information delivery,				
Prescribing doctor	– Verbal only	65 (98.5%)	40 (35.4%)	25 (28.7%)
	Written only	1 (1.5%)	1 (0.9%)	0 (0%)
	Both	0 (0%)	0 (0%)	0 (0%)
Dispensing pharmacist	– Verbal only	3 (1.5%)	1 (0.9%)	2 (2.3%)
	Written only	156 (78.0%)	83 (73.5%)	73 (83.9%)
	Both	41 (20.5%)	29 (25.7%)	12 (13.8%)
Language used is understandable and clear,				
Prescribing doctor		66 (100%)	41 (100%)	25 (100%)
Dispensing pharmacist		186 (93.0%)	107 (94.7%)	79 (90.8%)

**Table 3:** Understandability of the information written in the drug package, prescription card and clinic notes book

	**Number (%)**		
	**All adults**	**Males**	**Females**
Understandable information,			
Drug name	2 (1.0%)	1 (0.9%)	1 (1.1%)
Drug dose	110 (55.0%)	54 (47.8%)	56 (64.4%)
Frequency of administration	104 (52.0%)	54 (47.8%)	50 (57.5%)
Reason for inability understand drug information			
Illegible hand writing	28 (14.0%)	20 (17.7%)	8 (9.2%)
Inability to read English	108 (54.0%)	54 (62.1%)	54 (47.8%)

The mean total score (out of 60) for the drug knowledge section of the questionnaire was 25.8 ± 16.5 (males – 25.3 ± 16.7, females – 26.4 ± 16.3, p-NS). The mean scores for each component of drug knowledge were; name (out of 20) 8.5 ± 6.9, dose (out of 10) 2.4 ± 3.6, frequency (out of 10) 7.8 ± 3.2 and indication (out of 20) 7.2 ± 6.5, there was no significant gender difference in mean scores for each component. The score for knowledge about the name of the drugs (out of 100) was ≤ 50 in majority (n = 131, 65.5%), while knowledge about the dose of drug (out of 100) was even poorer, with 72.0% (n = 144) getting < 25 marks. Forty seven percent (n = 94) of patients scored < 25 (out of 100) for knowledge about indication of the particular drugs. However knowledge about frequency of drug was much better with 78.0% of patients getting scores > 50 (out of 100). The knowledge status of the study population was categorized by their cumulative score (out of 60). Majority of the study population had a ‘poor knowledge’ (< 20 marks) (n = 92, 46.0%), while 36.5% (n = 73) had ‘adequate knowledge’ (20 – 40 marks) and only 35 (17.5%) participants had ‘good knowledge’ (>40 marks) of prescribed medications.

The results of the binary logistic regression analysis using the dichotomous variable ‘Good Knowledge of Drugs’ (0 = No, 1 = Yes) as the dependent factor and other independent variables mentioned above are shown in Table 4. The overall model was statistically significant as determined by the likelihood ratio test (*χ*2 = 38.22, p < 0.05). The Cox & Snell R-Square and Nagelkerke R Square values were 0.634 and 0.845 respectively. Hosmer and Lemeshow goodness-of-fit statistic (*χ*2) was 24.34 and suggested that the model was fit for the given data. The results indicate that Secondary (OR: 1.53, 95% CI 1.32 – 1.74) and Tertiary levels (OR: 2.79, 95% CI 1.54 – 4.05) of education, perception of own illness as being Moderate (OR: 1.23, 95% CI 1.12 – 1.34) or Severe (OR: 1.70, 95% CI 1.27 – 2.21) and being educated by a doctor (OR: 1.69, 95% CI 1.40 – 1.98) significantly increased the odds of having a ‘Good Knowledge of Drugs’.

**Table 4:** Binary logistic regression analyses of factors predicting ‘Good Knowledge of Drugs’

	**Odds ratio**	**95% CI**	***p *value**
Age	0.99	0.95 – 1.04	NS
Female gender (0 = Male)	1.17	0.50 – 2.73	NS
Level of Education (0 = No formal education)			
Primary education	0.82	0.62 – 1.12	NS
Secondary education	1.53	1.32 – 1.743	<0.05
Tertiary education	2.79	1.54 – 4.05	<0.001
Monthly income (0 = No income)			
< Rupees^*^ 15,000	0.54	0.24 – 0.74	NS
≥ Rupees 15,000	0.93	0.83 – 1.03	NS
First Language being Tamil (0 = Sinhala)	0.74	0.60 – 0.88	NS
Perception of severity of illness (0 = Mild)			
Moderate	1.23	1.13 – 1.34	<0.05
Severe	1.70	1.27 – 2.21	<0.01
Educated by doctor (0 = No)	1.69	1.40 – 1.98	<0.05
Educated by pharmacist (0 = No)	0.65	0.45 – 0.91	NS

*NS* – Not significant, *- 1 US $ = Rupees 135.00.

## Discussion

Patient’s knowledge about prescribed medication is an important factor determining their compliance and ultimate outcome of a disease. The present study explored factors associated with poor knowledge on prescribed medications in patients with LEP in a resource-poor setting. Our results suggest that patients’ knowledge on drug information was unsatisfactory, particularly their knowledge on drug names, doses and indications.

Majority of the patients were unable to read and understand the information written in the DP, PC or CNB. The main reason for this was patients’ inability to read the information written in English. These results are comparable with previous reports from Sri Lanka investigating the language and its impact on reading information on labels, packages and leaflets in drugs and devices [18]. The doctor’s contribution towards educating patients on drug information (as assessed by the patients) was also unsatisfactory, as only 33.0% of patients were educated by the prescribing doctors. Furthermore, the binary logistic regression analysis shows that being educated by a doctor was significantly associated with having a ‘Good Knowledge of Drugs’. In the Asian setting studies have shown that limited time for consultations seems to be the main barrier limiting doctor-patient communication [26]. High patient loads at hospitals caused by ineffective health care systems and hesitancy by patients with low-levels of education to engage in communication also influences the doctor–patient interaction [26]. Hence, it is important to rectify these barriers in order to enhance doctor-patient communication and improve patients’ knowledge. However, it may be impractical to immediately overcome obstacles such as limited time for consultation and high patient loads at large teaching hospitals like the IC-NHSL. A plausible alternative could be to have specially trained nurse educators. Studies have shown that specialist nurse educators in heart failure patients improved patients’ knowledge and reduced risk of re-admission [27]. Furthermore, our results also show that pharmacist were involved in educating all patients on drug information, hence enhancing the abilities of dispensing pharmacists’ on patient education might help improve knowledge.

Patients’ perception about the disease and attitudes towards an illness are also known to affect their knowledge. Patients with negative attitudes towards their illness can be unwilling to follow advice in their management plans [28]. In addition positive illness perception was related to reflection/consideration when taking medications [29]. Majority of patients (61.5%) admitted that their knowledge on drugs were unsatisfactory and even a larger majority (70.0%) believed it is important to have a good knowledge about drugs that they are prescribed. Furthermore, more than 90% of the patients requested further information on drug names, dose, indications and side effects. Patients’ own perception about their illness as being moderate or severe was also significantly associated with a ‘Good Knowledge of Drug’. Previous studies have shown that patients’ own perception of their illness strongly affects compliance, behavioral control of risk factors and self-management [30-32]. Hence, there are reasons for advising health care personnel to try and take the patient’s perceptions of illness and medication into account during consultation.

Patient’s knowledge on prescribed medications was significantly associated with their level of education, while Secondary and Tertiary levels of education were significantly associated with ‘Good Knowledge of Drugs’. It is possible that higher levels of education were associated with a higher proficiency in English since it is taught as a second language throughout the school curriculum in Sri Lanka [33]. Although improving patients’ level of education is not a feasible intervention in adult patients attending IC-NHSL, it serves as a possible mean of early recognition of patients with poor knowledge in order to apply selective interventions aimed at enhancing knowledge. Studies done in Sri Lanka have shown that including health related information in discharge summaries in native language improves patient knowledge of their illnesses and medication [34]. Hence, it may be plausible to include prescribing information also in the native language in order to gain a similar beneficial effect in improving knowledge on prescribed medications.

The present study has several limitations. We evaluated the patients’ subjective perception of severity of their own illness; however we do not have the data to show the accuracy of perceived severity. Furthermore other observations such as adequacy and clarity of information given by doctors/pharmacist are also as perceived by the patients. In addition, the study was limited to patients with cardio-vascular disease. However since majority of non-communicable chronic diseases affecting Sri Lankans such as diabetes, hypertension, obesity and cerebrovascular disease share common risk factors and management principals, the results of our study could also be generalized to these diseases. In addition, we utilized an expert validated scale for measuring medication knowledge in the absence of a statistically and culturally validated tool applicable to the local population. The patient’s knowledge on prescribed medications was based on memory and may not reflect the ability to use medications at the home setting in the presence of reference material such as prescription books and notes.

## Conclusions

In a resource-poor setting in patients with Limited English Proficiency, lower level of education and misperception of illness severity resulted in reduced knowledge on prescribed medications. Being educated by doctors significantly improved patients’ knowledge, however the doctors’ contribution at present to deliver quality health information for their patients was are not satisfactory. Hence, health care professionals should pay extra attention when treating patients with Limited English Proficiency.

## Competing interests

The authors declare that they have no competing interests.

## Authors’ contributions

MHMTSP, SAUP, SNP, GRC, and SJ made substantial contribution to conception and study design. MHMTSP, SAUP and SNP were involved in data collection. GRC, SJ, PR and AMMC were involved in refining the study design, statistical analysis and drafting the manuscript. PR and AMMC critically revised the manuscript. All authors read and approved the final manuscript.
